# Osseous differentiation in cystosarcoma phyllodes - diagnosed by fine needle aspiration cytology

**DOI:** 10.4103/0970-9371.73305

**Published:** 2010-10

**Authors:** Jayashree Krishnamurthy

**Affiliations:** Department of Pathology, VIMS, Bellary, India

**Keywords:** Cystosarcoma phyllodes, phyllodes tumor, Osseous differentiation, fine needle aspiration cytology

## Abstract

Osseous differentiation within a phyllodes tumor is extremely rare. Cytological and histological findings of a case of malignant phyllodes tumor with osseous differentiation are presented. A 45-year-old female had a malignant phyllodes tumor with osseous stroma diagnosed by fine needle aspiration cytology. The cytological findings were representative of the histological features. The diagnosis of these tumors preoperatively is important in planning the most appropriate treatment. It is also important to follow up these patients postoperatively for long periods for recurrence and metastasis.

## Introduction

Phyllodes tumor – cystosarcoma phyllodes – is an uncommon mammary tumor with heterogenous histology consisting of both epithelial and stromal components. The cellularity of its connective tissue component is the most important feature distinguishing it from fibroadenoma. A small percentage of tumors are histologically overtly malignant, constituting less than ten percent of all phyllodes tumors.[1] Osseous differentiation is an extremely rare stromal alteration in phyllodes tumor causing histologic complexity. Preoperative diagnosis of these tumors by fine needle aspiration cytology (FNAC) and identification of overtly malignant tumor can help in planning the extent of surgery.

A case of phyllodes tumor with osseous differentiation is reported.

## Case Report

A 45-year-old woman presented with slowly progressive palpable mass of seven years duration in the upper outer and inner quadrant of the left breast measuring 10×6×5 cm, It was non-tender, mobile, not fixed to the skin or the underlying chest wall. It was of variable consistency with cystic firm and hard areas. There was no nipple retraction or enlarged lymph nodes. The general physical and systemic examination was within normal limits.

The routine investigations were done and found to be normal. Fine needle aspiration of the lesion was performed.

The cytological evaluation suggested a phyllodes tumor (high grade) with osseous stroma. Simple mastectomy was performed and the histologic diagnosis was malignant phyllodes with osseous differentiation.

**Figure 1:**
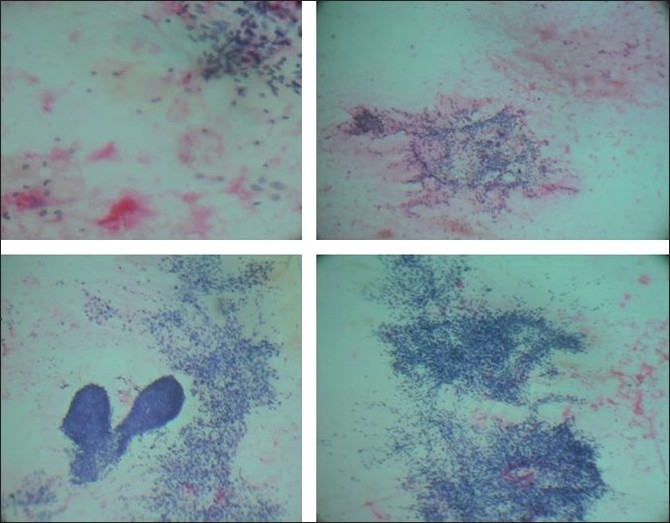
Smears showing tightly cohesive clusters of benign ductal epithelial cells and leaf-like stromal fragments with scattered osteoid in the background (H and E, ×100)

**Figure 2:**
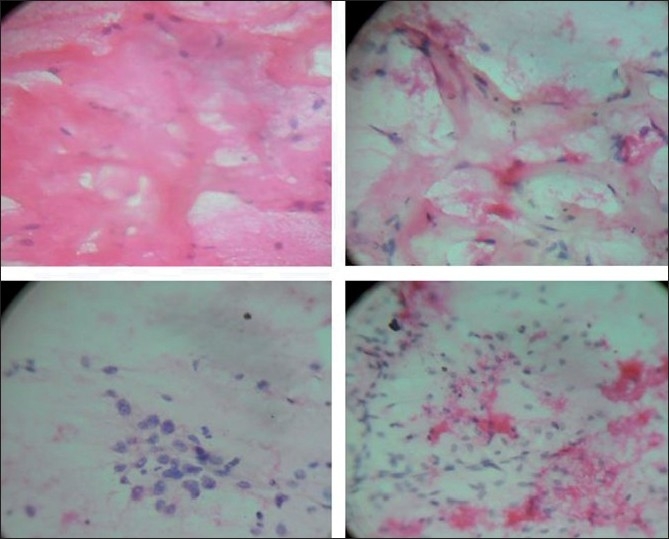
Smears showing amorphous and dense osteoid matrix along with scattered pleomorphic mesenchymal cells (H and E, ×400)

Cytological evaluation of hematoxylin and eosin stained smears revealed a cellular aspirate containing epithelial and stromal fragments [Figure 1]. The epithelial components consisted of frond like groups of cohesive ductal epithelial cells resembling those seen in benign tumors. The cells within the groups were uniform with round to oval nuclei, fine chromatin and scant to moderate cytoplasm. The stromal component that predominated the smears consisted of leaf-like stromal fragments within a myxoid stroma. Individual mesenchymal cells with a spindle-shaped nucleus and occasional stromal fragments showed capillary vessels traversing over them were also seen. The spindle-shaped cells showed nuclear atypia. Amidst stromal fragments were seen eosinophilic amorphous and finely fibrillar material which represented the osseous stroma. It was also seen as thin intracellular strands within a cell cluster resembling osteoblasts with eccentrically placed round nucleus with central nucleolus and dense cytoplasm [Figure 2]. A cytological diagnosis of phyllodes tumor (high grade) with osseous stroma was suggested.

A simple mastectomy was performed and the specimen was subjected to histopathological examination.

**Figure 3:**
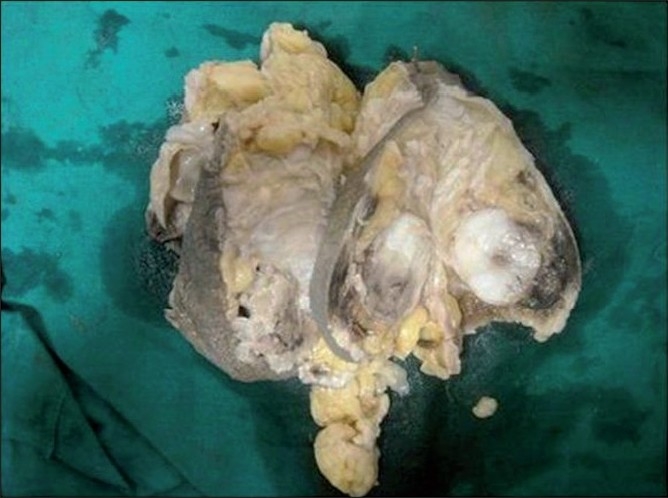
Cut section of mastectomy specimen showing cystic, solid, and osseous areas

Grossly the mastectomy specimen was covered by an elliptical bit of skin with nipple and areola measuring 12×12×8 cm. An irregular cystic mass was seen 2 cm from the nipple measuring 5×4 cm, which drained 10 ml of blood mixed fluid. Cut section through the nipple revealed an ill-defined grey white mass showing a variegated appearance with cystic, friable and solid areas. Adjacent to this was seen a well-defined hard glistening region 4 cm in diameter [Figure 3].

**Figure 4:**
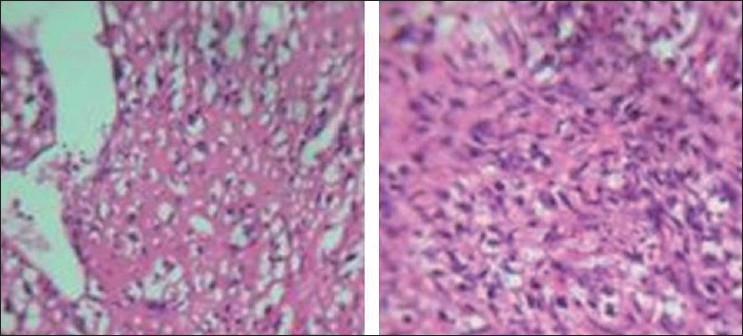
Sections showing sarcomatous stroma and osseous stroma with benign epithelial ductal component (H and E, ×400)

Microscopically the tumor was composed of a biphasic pattern consisting of epithelial and stromal fragments. The ductal components were benign. The stromal elements were predominant, arranged in interlacing fascicles and whorls. The cells showed marked pleomorphism, cellular atypia, multinucleated forms and abnormal mitosis [Figure 4]. Areas of osteoid matrix with osteoblasts were seen amidst the pleomorphic stromal components [Figure 4]. The final diagnosis of malignant phyllodes tumor with osseous differentiation involving the left breast was made.

## Discussion

Phyllodes tumors of the breast are rare neoplasms accounting for less than 1% of breast tumors, 2–3% of mammary fibroepithelial neoplasms and 1.5% of all breast carcinomas.[1,2] Malignant phyllodes tumors are rare ones comprising 0.18% of all breast malignancies.[3] They occur most often in 30–70 year-old females, the median age at the time of diagnosis being 45 years with a few occurring in adolescents.[4] These tumors present as discrete palpable masses with rapid enlargement. They are characterized by a tendency for local recurrence and extreme rarity of a distant metastasis. The phyllodes tumor is a tumor of specialized mammary stroma with a capacity for epithelial induction.

The value of FNAC in the diagnosis of phyllodes tumor is variable and the accuracy ranges from 25 to 70%.[5] A cytological diagnosis of phyllodes tumor is suggested by the presence of epithelial and stromal elements, the stroma presenting as cellular leaf-like fragments and isolated mesenchymal cells.[6] The stromal overgrowth is an important criterion for the diagnosis of malignancy in phyllodes tumor.[8] The parameters suggesting malignancy are extreme paucity of epithelial elements, and stromal cells in diffuse sheets and less cohesive clusters with marked stromal atypia and mitotic activity.

The cytological smears from phyllodes tumors share many similarities with those of fibroadenoma causing difficulty in diagnosis. The presence of large cells low epithelial / stromal ratio, benign nature of epithelial elements and stromal atypia favors a diagnosis of phyllodes tumor.[5]

Cystosarcoma phyllodes can have heterologous stromal components consisting of bone, cartilage, smooth muscles or fat.[8] The neoplastic stromal components is highly pleomorphic reminiscent of fibrosarcoma, malignant fibrous histiocytoma, rhabdomyosarcoma liposarcoma, and osteogenic sarcoma.[1,9,10] Osseous differentiation of the stroma contributes to the histologic complexity of these tumors and correlate with malignant phyllodes tumor (high grade).

## Conclusion

Malignant phyllodes tumor of the breast can be diagnosed by FNAC. It is very important to acknowledge the morphologic variants of sarcomatous stroma and to recognize the cytologic features of such rare tumors that help in planning the most appropriate treatment. It is also important to follow up the patients postoperatively for long periods for recurrence and metastases.
